# Is It worth Considering Circulating microRNAs in Multiple Sclerosis?

**DOI:** 10.3389/fimmu.2016.00129

**Published:** 2016-04-05

**Authors:** Ferdinand Jagot, Nathalie Davoust

**Affiliations:** ^1^Biology Department, Ecole Normale Supérieure de Lyon, Université de Lyon, Lyon, France; ^2^Laboratory of Molecular Biology of the Cell, UMR5239 CNRS/Ecole Normale Supérieure de Lyon, UMS 344 Biosciences Lyon Gerland, Université de Lyon, Lyon, France

**Keywords:** multiple sclerosis, relapses, circulating miRNAs, extracellular vesicles, inflammation, demyelination, exosomes, biomarkers

## Abstract

New evidence has highlighted that miRNA production and trafficking can be dysregulated in both autoimmmune and neurological disorders. Multiple sclerosis (MS) in particular is an autoimmune pathology leading to neurodegeneration. Profiling studies performed on cells derived from MS patients have described a dysregulated network of miRNAs in both immune and neural cells. Interestingly, new evidence has emerged showing that circulating miRNAs are also dysregulated in MS body fluids, including plasma/serum and cerebrospinal fluid. This review summarizes the current scientific theories on the function of this altered circulating miRNA network. It builds up new insights about miRNA transfer mechanisms including extracellular vesicle trafficking involved in cell-to-cell communication and the possible physiopathological functions of these transfers in MS. Finally, this review proposes that monitoring altered miRNA expression levels could serve as a potential biomarker read-out of MS subtype and severity.

## Introduction

Multiple sclerosis (MS) is a human lymphocyte-mediated autoimmune disease affecting the central nervous system (CNS). Neurodegeneration of the CNS is associated with both neuron demyelination and inflammation in the so-called active lesions. It is common to separate relapsing–remitting MS (RRMS), in which periods of relapses (attacks) alternate with periods of remission, from primary progressive MS (PPMS) characterized by a constant worsening of CNS condition and minor remissions. In about 60% of RRMS cases, the disease secondarily turns into a progressive form (secondary progressive or SPMS). miRNAs are small non-coding RNAs of about 20 nucleotides inhibiting the expression of their mRNA targets through degradation and/or by stopping mRNA translation (1). In most cases, they are transcribed by polymerase II into a stem–loop primary miRNA (pri-miRNA). The pri-miRNA is cleaved in the nucleus by a complex, including the RNase III Drosha, forming a pre-miRNA, which is exported into the cytosol thanks to Exportin 5. The cytosolic pre-miRNA is then cleaved by the endoribonuclease Dicer and associates with the RNA-induced silencing complex (RISC), which mediates mRNA translational silencing/degradation [reviewed in Ref. (2)].

The expression of miRNAs in MS was first assessed in cells derived either from MS patients’ blood samples or from active lesions (3, 4). Interestingly, a deregulated expression of certain cellular miRNAs has been proposed as a putative trigger in MS physiopathology (3, 5). In particular, miRNA regulation of disease-associated proinflammatory lymphocyte Th17 differentiation has been actively investigated (3, 5–7). miR-21, miR-20b, and miR-326 were shown to regulate Th17 differentiation by modulating the expression of pivotal transcription factors of T cell differentiation [SMADs (6), STATs (7), RORγ (7), and Ets-1 (3)]. In this context and as miRNAs initially identified as cellular can be dysregulated in circulating fluids as well, interesting questions are raised regarding the potential functions of circulating miRNA in MS physiopathology. The expression of circulating miRNAs in body fluids, including serum and cerebrospinal fluid (CSF), of MS patients was indeed investigated in recent studies (8). In this review, “circulating miRNAs” refers to all cell-free miRNAs.

Using a combination of established methods of miRNA profiling, it is now possible to propose a provisional network of dysregulated miRNAs in MS. These approaches provide new insights and raise some interesting issues: how are disrupted cellular and circulating miRNA networks related together? What could be the role of circulating miRNAs in MS physiopathology? Which molecular vectors carrying circulating miRNAs would be the most relevant to study and could these vectors be miRNA-associated actors of MS pathogenesis? This review first recapitulates the up-to-date network of dysregulated circulating miRNAs in MS patients. Second, extracellular vesicles (EVs) are considered as miRNA vectors and are proposed to be involved in miRNA-mediated pathogenesis. Finally, circulating miRNAs are proposed to be convenient, reliable, and accurate biomarkers to differentiate MS subtypes and evaluate MS severity.

## Review

### Dysregulated Network of Circulating miRNAs in MS Patient Fluids

Junker and colleagues have described the first miRNA profiling analysis performed on MS patient brain lesions in 2009 (4). Exponential number of publications was consecutively released focusing on miRNA profiling on different blood fractions, including immune cells and plasma fluid. In this review, the term “plasma” will be used interchangeably to describe serum and/or plasma. It aims at simplifying the view of circulating miRNAs, although serum and plasma certainly have different miRNA contents.

The main approach of miRNA profiling analyses is based on a microarray analysis in combination with quantitative PCR. But because the process of RNA extraction toward profiling is not standardized, caution is required when comparing two different analyses. Results strongly depend indeed on RNA extraction, miRNA quantification, and interpretation strategies mainly based on the kind of internal control and statistical analysis that have been used. For instance, dealing with miRNA quantification, two important studies used the miRCURRY LNA™ Universal RT microRNA PCR kit from Exiqon (9, 10). It is based on SYBR green incorporation during qPCR performed in 384 well plates, each containing different miRNA-specific primers. On the other hand, miRNA quantification by Siegel and colleagues (11) was based on the incorporation of aminoallyls in mRNAs produced *via* the transcription of miRNA-derived cDNA (Amino Allyl MessageAmp™ II aRNA amplification kit from Life Technologies). Such differences in the procedure for miRNA profiling lead to massive variability. Researchers now aim at standardizing these techniques to comprehend profiling metadata (12). Interestingly, a next-generation sequencing (NGS) technique was recently used to assess miRNA dysregulation in MS patients and confirmed the microarray analyses showing identical regulation of the eight miRNAs, which were previously found to be dysregulated (13). A previous study using the NGS technique also identified 43 miRNAs that were dysregulated in immune-activated lymph nodes of experimental autoimmune encephalomyelitis (EAE)-susceptible rats (14). In NGS technologies based on RNA sequencing (RNA-seq), the read counts of each miRNAs allow to estimate their relative expression level. RNA-seq has been shown to provide results with higher sensitivity and broader dynamic range as compared to microarray analyses (15, 16). Nevertheless, microarrays are still the most common technique to conduct miRNA profiling experiments for both financial and practical reasons. NGS outputs are massive and lack standardized/user-friendly pipelines for processing and analyzing the data (17). However, as sequencing nucleotides gets cheaper and as new pipelines are being developed, we can expect NGS to become the predominant tool for monitoring miRNA levels.

**Figure 1:**
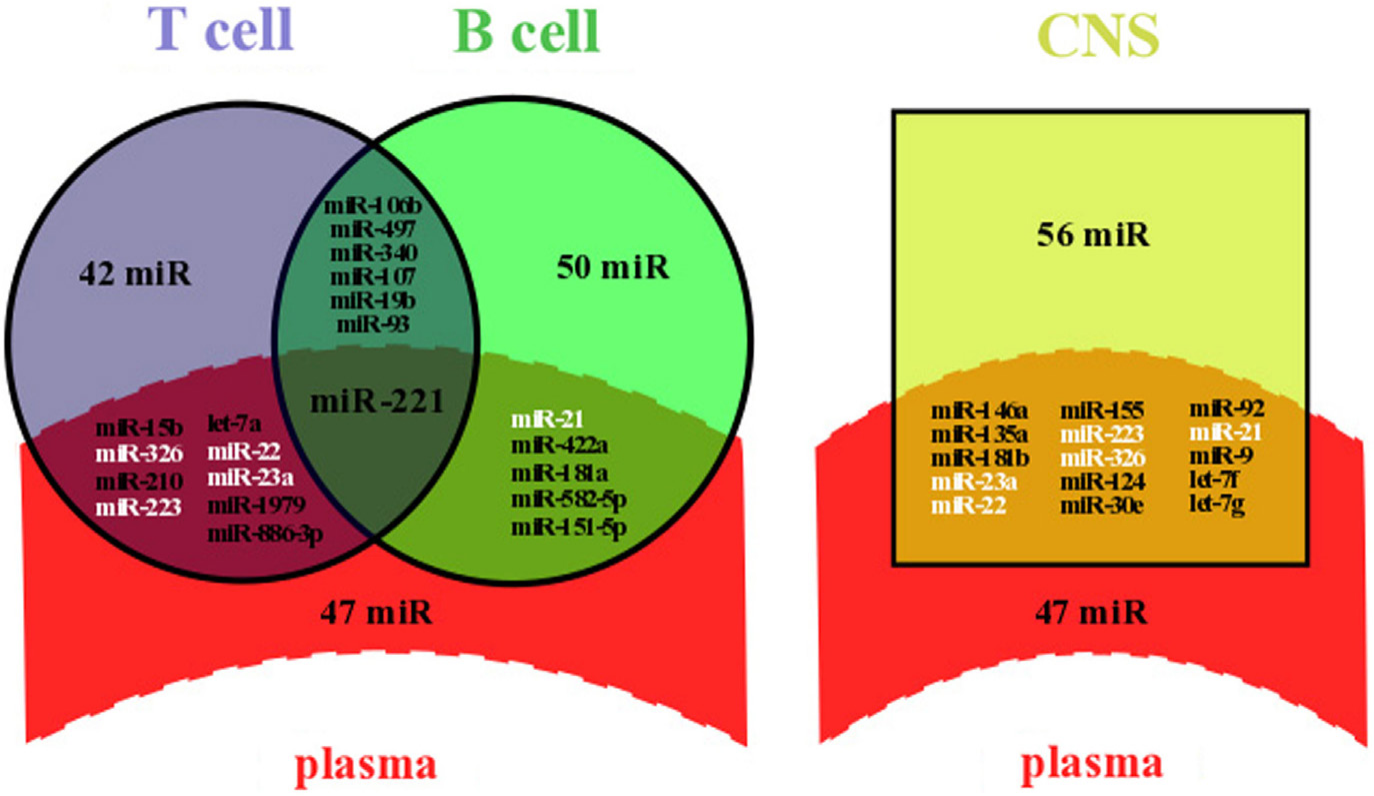
**Overlapping between dysregulated miRNAs in plasma and lymphocytes or in plasma and the CNS of MS patients**. Dysregulated miRNAs from plasma and lymphocytes (left panel) or from plasma and the CNS (right panel) were either identical (overlapping area) or not (single area). Data were compiled from miRNA profiling studies performed on plasma (5, 9–11, 18), immune cells [B (20–22) or T cells (3, 22–26)], and the CNS (astrocytes, oligodendrocytes, brain endothelial cells, whole brain lesions, and whole brain) (4, 27–30). Briefly, we selected dysregulated miRNAs from microarray profiling studies, filtered out miRNAs with non-significant variation of the expression level, and highlighted commonly dysregulated miRNAs. All overlapping miRNAs are listed and those written in white color are dysregulated in at least three compartments, including plasma and the CNS; miR, miRNA.

Microarray analyses of MS patients’ whole blood (plasma and cells) or plasma have been extensively used to assess miRNA expression levels (5, 9–11, 13, 18, 19). As a result, a significant amount of data about circulating miRNA dysregulation in MS patients compared to healthy controls has been generated, supplementing an already complex dataset of dysregulated miRNAs in immune cells and in the CNS. We applied a systematic search of miRNAs that have been shown to be deregulated in plasma (5, 9–11, 18), immune cells [B (20–22) or T cells (3, 22–26)], or the CNS (astrocytes, oligodendrocytes, brain endothelial cells, whole brain lesions, and whole brain) (4, 27–30). As a result, we collected 19 studies that had generated microarray profiling metadata and filtered out the miRNAs that were not significantly deregulated in these microarrays. The significance of miRNA dysregulation is based on the statistical tests performed in the microarray studies themselves. We then compared the list of dysregulated miRNAs in each compartment side by side and highlighted the commonly dysregulated miRNAs between immune cells and plasma or between the CNS and plasma (Figure 1).

Our systematic search revealed that at least 62 miRNAs have already been shown to be significantly deregulated in plasma of MS patients, among which a majority was upregulated (54  miRNAs). A significant number of miRNAs were commonly dysregulated between plasma and immune cells (15 over a total of 160 miRNAs) and between plasma and the CNS (15 over a total of 118 miRNAs) (Figure 1). The role of such a diagram is to draw attention on some miRNAs for further comprehensive and functional analyses. The finding of specific miRNAs deregulated in several compartments could help deciphering the compartment-specific role of miRNAs and help finding the interactions between these compartments. Interestingly, miR-221 is upregulated in both plasma (9) and Treg cells (24), whereas miR-221 is downregulated in B cells (20) (Figure 1). In mature dendritic cells (DCs), miR-221 upregulation has been associated with increased levels of p27^kip1^ driving apoptosis (31). Also, miR-221 upregulation in T cells has been shown to inhibit survival and proliferation (32).

We suggest that commonly dysregulated miRNAs should be prioritized for functional assays in a cell-specific context. For instance, miR-23a, miR-223, miR-22, miR-326, and miR-21 expressions are altered in at least three different compartments, including plasma and the CNS (Figure 1). Additional evidence to select putative miRNAs involved in MS pathogenesis include (i) the degree of deregulation observed (e.g., fold change as compared to the control), (ii) a validation by RT-qPCR, and (iii) at least one demonstrated mRNA target. Thus, focusing on mi-326 would be of particular interest, as its expression is drastically altered in CD4^+^ T cells (eightfold) (3), in active brain lesions (ninefold) (4), and in plasma (twofold) (5) and as it has been shown to target Ets-1, a negative regulator of Th17 differentiation (3). By contrast, although miR-22 expression is altered in three different compartments, it is with low fold change (4, 11, 24) and miR-22 has no defined but only predicted targets, including the B cell translocation gene 1 (BTG1), a regulator of cell proliferation, and the estrogen receptor alpha (ESRα) (11).

Finding the miRNAs that are differentially expressed in different compartments enables to prioritize miRNAs for functional involvement in MS pathogenesis, but it also aims at understanding the functional distribution of a given miRNA in different compartments. It remains a complicated task since among the 30 miRNAs commonly dysregulated in plasma and another compartment, about one-third were upregulated in one compartment when downregulated in another one. miR-15a, a putative trigger of the regulation of CD4^+^ T cell apoptosis (23), well illustrates the complexity of miRNA dysregulation: miR-15a has been highlighted downregulated in whole blood (19), B cells (20), CD4^+^ T cells (23), and brain endothelial cells from brain lesions (27) but upregulated in regulatory T cells (24) and active brain lesions (4). It remains unclear why the same miRNA would be differently deregulated between plasma and another compartment. This observation could be assigned to a lack of standardization in RNA extraction and quantification protocols leading to heterogeneous results. But one can speculate that a difference in miRNA levels between the inner (cellular) and outer part (extracellular) of the cell is a result of a selective process of miRNA release. In fact, major hypotheses involve apoptosis or mechanisms driving miRNA release into circulation. The latter will be further discussed considering the mechanisms triggering miRNA release.

### Extracellular Vesicles as Potential Vectors for Circulating Dysregulated miRNAs

**Figure 2:**
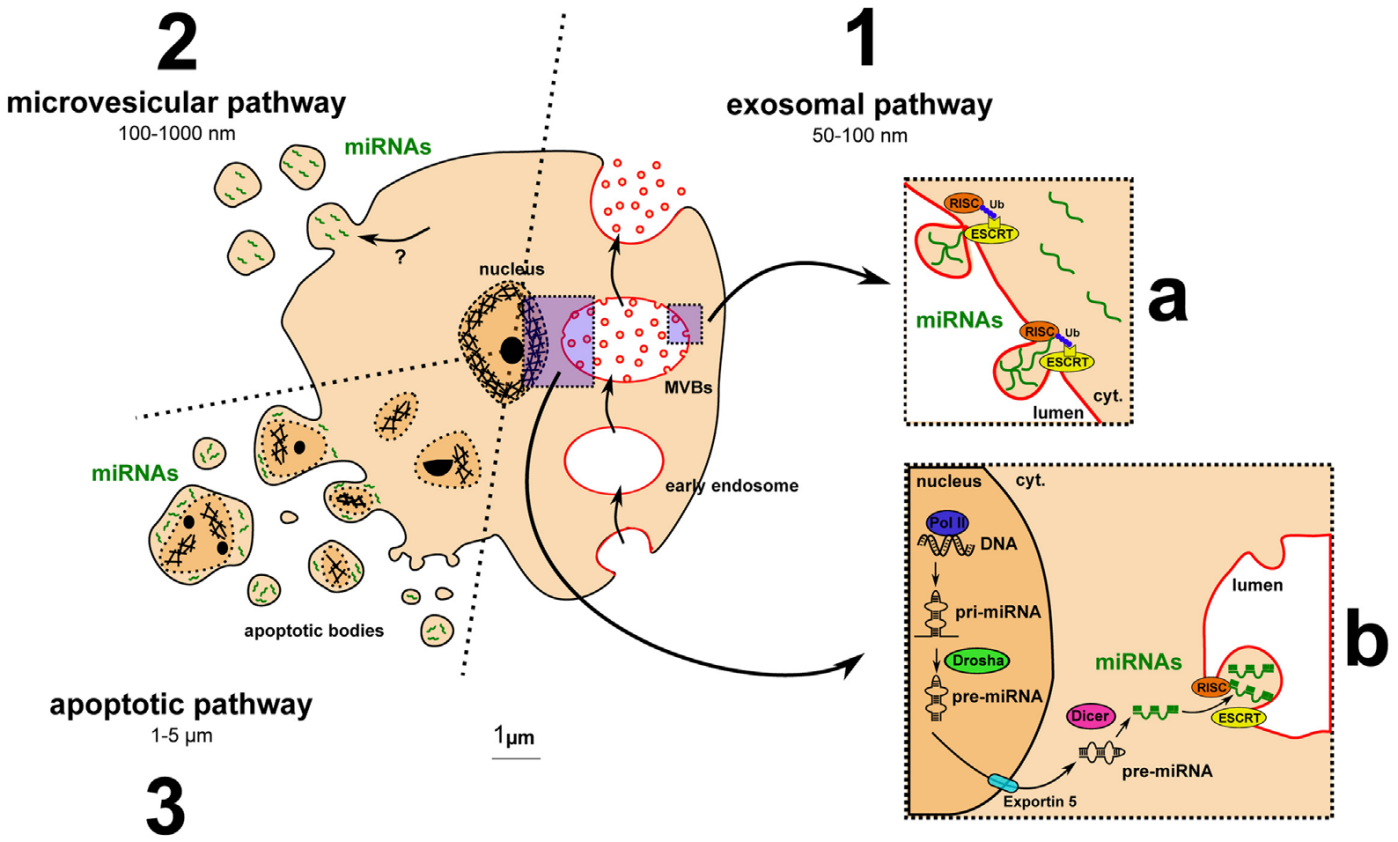
**Major pathways for extracellular vesicle biogenesis and for miRNA incorporation into exosomes**. (1) The exosomal pathway includes MVB formation, exosome budding into MVB lumen, and exocytosis-mediated exosomal release. (a) Ubiquitinated RISC- and ESCRT-dependent incorporation of miRNAs into exosomes: model from Gibbings and colleagues (35). (b) Main pathway of miRNA biogenesis detailed in the Section “Introduction.” (2) The microvesicular pathway: miRNA uptake by microvesicles remains to be clarified. (3) The apoptotic pathway: apoptotic cells release apoptotic bodies containing fragment of the nucleus and putative miRNAs.

The question of how dysregulated miRNAs are carried into MS patient fluids has not been addressed yet. However, extracellular miRNAs have already been identified with different carriers, including EVs (33). EVs can be distinguished based on their origin, size, and membrane markers. Among the different type of EVs, miRNAs were shown to be carried by exosomes, microvesicles (MVs), or apoptotic bodies mainly (Figure 2). Exosomes range between 50 and 100 nm, similar in size to viral or lipoprotein particles. They are formed by budding into the lumen of multivesicular bodies (MVBs), important intermediates in the endolysosomal pathway, and are released by exocytosis of MVBs. Exosomes display different RNA profiles compared to cellular ones, suggesting a selective and active incorporation of miRNAs (34). A recent study highlighted that this selective and active incorporation of miRNAs into exosomes could be dependent on RISC and endosomal sorting complexes required for transport (ESCRT) (35). RISC and ESCRT are canonically involved in miRNA-mediated translation inhibition and budding processes, respectively. Further investigation is required to determine how these pivotal complexes regulate miRNA release and whether this regulation could drive differences between cellular and extracellular miRNA levels. The second category of EVs is MVs, which are 100–1000 nm particles directly budding from the plasma membrane. miRNAs are also found in MVs (36), but the mechanism by which they are incorporated is not yet elucidated. Apoptotic bodies are often much larger, from 1 to 5 μm. Methods to isolate and characterize them are lacking, and most studies perform coculture with apoptotic cells (33). Recent data, however, suggest that miRNAs can be carried by endothelial cell-derived apoptotic bodies (37).

Otherwise, some studies highlighted protein-associated circulating miRNAs. Arroyo and colleagues described human blood extracellular miRNAs associated with a RISC unit called Ago2 (38). It is premature to draw conclusions about knowing whether this observation is physiological or not. Indeed, an active mechanism of protein-associated miRNA release has not been described yet. These miRNA–Ago2 complexes could also derive from dead cells or derive from an artifact through vesicle degradation during miRNA purification. In fact, it is known that miRNA–Ago2 complexes are found in exosomes (8). Depending on culture conditions and cells used, extracellular miRNAs have also been described associated with RNA-binding protein nucleophosmin (NPM1) (39) or bound to HDLs (40). Mechanisms by which the release is possible still remain unknown.

Evidences are in favor of EVs for being the main vectors of circulating miRNAs. Indeed, mechanisms of miRNA release into EVs have been largely described, whereas pathways leading to extracellular protein-associated miRNAs are poorly understood. But how miRNA-associated EVs could be implicated in MS pathogenesis? First, EVs were shown to be upregulated in the blood of MS patients compared to healthy controls (41). Thus, a difference in EV production could participate in establishing the dysregulated network of circulating miRNAs. Second, EVs have been implicated in MS pathogenesis (42), without a demonstrated role of miRNAs, but through associated protein factors that mediate brain blood barrier (BBB) disruption. And finally, EVs are currently emerging as critical actors in cell-to-cell communication. It appears that a complex network of circulating miRNAs actually coexist with a complex network of EVs. Emerging results unveil that many different cells produce different types of EVs (Table 1). Among these cells, B cells, T cells, and neural cells were shown to be involved in MS pathogenesis. It was highlighted that B cells, Th1, Th17, regulatory T cells, brain endothelial cells, and monocytes produce not only exosomes but also MVs. DCs, B and T cells, and monocytes were shown *in vitro* (by coculture or EV addition) or sometimes *in vivo* to be recipient for EVs. In the CNS, neural cells, including glia cells, also exchange EVs (referenced in Table 1). Recently, immune cells were shown to transfer genetic information to brain cells *in vivo* (43). This transfer is likely to happen through EVs as purified EVs contained the genetic probe. Moreover, this genetic transfer was increased under peripheral inflammatory conditions. Peripheral inflammation and BBB disruption in MS pathology are two factors that could explain a passage of EVs from the CNS to the blood. It is also of interest to establish whether immune cells invading the CNS of MS patients could produce, locally in the CNS, EVs.

**Table 1:** **Emerging network of extracellular vesicles involving immune and brain cells as producing and recipient cells**.

EV producing cells	Type of EVs	EV receiving cells	*In vivo*	*In vitro*		Reference
				EV^a^	CoC^b^	
EBV-infected B cells	Exosome	Monocyte-derived DC	Yes	Yes	Yes	(34)
B cells	Exosome	No	No	No	No	(44)
CMV-infected endothelial	Exosome	Blood CD4^+^ T cells	No	Yes	Yes	(45)
Brain endothelial cells	Microvesicle	Monocytes	No	Yes	No	(46)
GM-CSF-induced DCs	Exosome	CD8^+^ T cells	No	Yes	Yes	(47)
Migratory DCs	Not defined	Lymph node-resident CD8^+^ T cells	Yes	No	No	(48)
GM-CSF-induced DCs	Microvesicle	No	No	No	No	(49)
Blood T cells	Exosome	No	No	No	No	(50)
Regulatory T cells	Exosome	T and B cells, DCs	No	Yes	Yes	(44)
Th1 and Th17 cells	Exosome	No	No	No	No	(44)
THP-1 monocytic cells	Microvesicle	No	No	No	No	(51)
Microglia	Microvesicle	Hippocampal neurons	No	Yes	No	(52)
Oligodendrocyte	Exosome-like	Oligodendrocyte	No	Yes	No	(53)
Astrocyte	Exosome	Spinal neurons	No	Yes	Yes	(54)

*^a^Direct addition of EVs on recipient cell*.

*^b^Cellular coculture (CoC) between producing and recipient cells*.

*Colors depict: 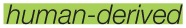, 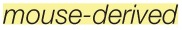, and 
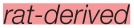
 cells*.

It has been demonstrated that many miRNAs are functionally involved in MS pathogenesis when their expression is altered. Determining the role of circulating miRNAs requires considering their carrier. In the following section, we propose cellular mechanisms describing the carrier-dependent effects of some circulating miRNAs on MS pathogenesis.

### Potential Role of Circulating miRNAs in MS Pathogenesis

**Figure 3:**
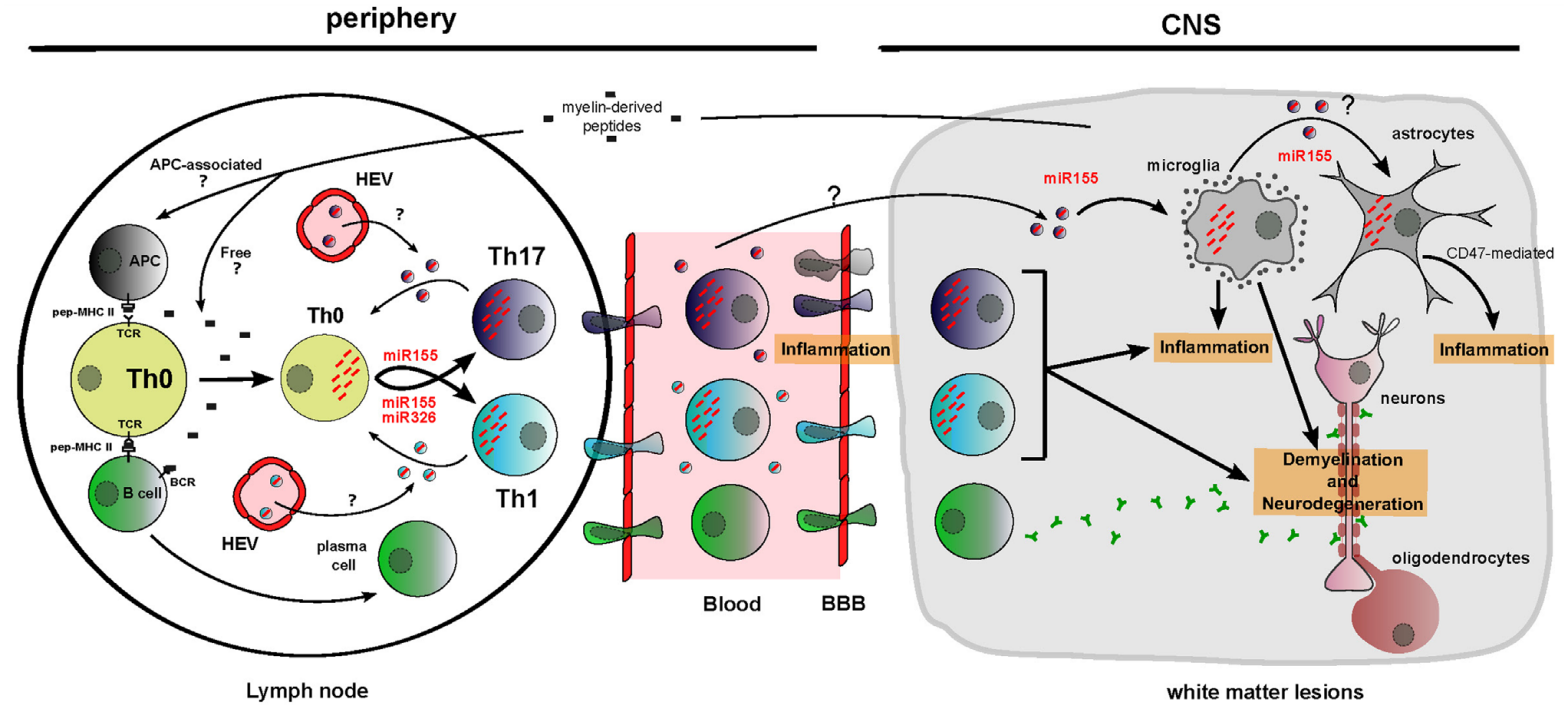
**Functions of dysregulated cellular and circulating miRNAs in MS/EAE physiopathology**. Myelin-derived peptides reach the lymph node as free autoimmune-prone antigens or associated with antigen-presenting cells. It results in B cell and T cell activation and migration of these cells across a porous brain blood barrier. Unbalanced differentiation toward proinflammatory T cell subsets Th1 and Th17 is amplified by cellular miR-155 and miR-326 upregulation and by cell non-autonomous transfer of these miRNAs between T cells through putative EVs. In the CNS, miR-155 participates to microglia-mediated inflammation/neurodegeneration. Brain inflammation is also aggravated by miR-155-mediated decrease of CD47, driving vulnerability of neural cells (evidence being for astrocytes) toward microglia-mediated phagocytosis. miRNAs are depicted as red dashes. EVs appear in the color of the producing cell and contain a single effective miRNA for simplification. HEV, high endothelial venules.

Experimental autoimmune encephalomyelitis is a mouse (sometimes monkey or rat) autoimmune model aiming at reproducing MS pathology features. It is now accepted that in both MS and its EAE model, pathogenesis involves lymphocytes including B cells (55), Th17 and Th1 cells (56) specific to myelin antigens. Production of these myelin-specific lymphocytes should require a primary migration of myelin-associated DCs and transfer of free antigens from the CNS to peripheral lymph nodes leading to the Th1/Th17 and B cell response, respectively (Figure 3). Migration of these cells into the CNS through a porous BBB participates to both inflammation in active lesions and axon demyelination.

The implication of cellular miRNAs in MS and EAE pathogenesis is currently strongly investigated. In order to define the new concepts about circulating miRNA function, it is necessary to consider different elements: (i) producing and recipient cells for EVs, (ii) cellular and extracellular miRNA dysregulation, and (iii) cellular miRNAs functionally involved in MS/EAE. Some miRNA function and distribution seem relevant to suggest circulating miRNA implication in Th17-mediated, Th1-mediated, and brain-resident cell-mediated MS pathogenesis (Figure 3).

#### miR-326

miR-326 is upregulated in CNS active lesions (4) and circulating CD4^+^ T cells (3) of MS patients. A study from Du and colleagues (3) on EAE mice demonstrated that miR-326 targets Ets-1, a negative regulator of Th17 differentiation, thus promoting Th17 differentiation. Their *in vivo* experiments highlighted that downregulation of miR-326 leads to an improved EAE score through inhibition of Th17 differentiation, whereas miR-326 overexpression drives to a more severe EAE through Th17 differentiation. Besides, it is known that Th17 produce exosomes (44), T cells receive EVs (Table 1) and that circulating miR-326 is upregulated in MS patients (5). Thus, it may be worth considering a cell non-autonomous effect of circulating miR-326: Th17 cells may indeed produce exosomes containing increased amount of miR-326 driving an amplification of Th17 differentiation through EV transfer between T cells (Figure 3).

#### miR-155

Of most studied miRNAs, miR-155 is a master miRNA implicated in MS and EAE, because its action is on both immune and brain cells. Indeed, miR-155^−/−^ mice display a defective T cell development and are resistant to EAE (57). miR-155 promotes Th17 and Th1 differentiation in EAE mice leading to inflammation and demyelination (5, 57). Moreover, miR-155 has been shown to be upregulated in MS patient plasma (5). As Th1 and Th17 produce exosomes (Table 1) (44), a cell non-autonomous effect of circulating miR-155 could also amplify Th1 and Th17 differentiation thus increasing MS pathogenesis (Figure 3). Moreover, several studies have demonstrated that miR-155 is upregulated in MS brain white matter lesions (4), especially in microglia (28). miR-155 upregulation in microglia promotes its activation, which is detected in part through proinflammatory cytokine secretion (28). miR-155 upregulation in active lesions of MS patients also correlates with a decreased level of CD47, a transmembrane protein, which has been shown to dampen microglia activation, acting as a “don’t eat me” signal when induced on microglia neighboring cells (4). *In vitro*, CD47 has been highlighted as a target for miR-155 (4). Thus, miR-155 upregulation drives macrophage activation intrinsically but also indirectly by reducing CD47 expression in astrocytes and oligodendrocytes (4). This process would then be partially responsible for inflammation and neurodegeneration through phagocytic processes (Figure 3). Eventually as microglia, astrocytes and oligodendrocytes produce EVs (Table 1), one could consider that upregulation of miR-155 in one of these cell types could be sufficient to upregulate miR-155 in the other cells in a cell non-autonomous manner (Figure 3). However, these results were obtained for single miRNA analyses. Combining functional effects of miRNAs increases the complexity to interpret results. In fact, one study highlighted that regulatory T cells produce exosomes containing both miR-155 and let-7d that may be captured *in vitro* by Th1 cells (44).

**Figure 4:**
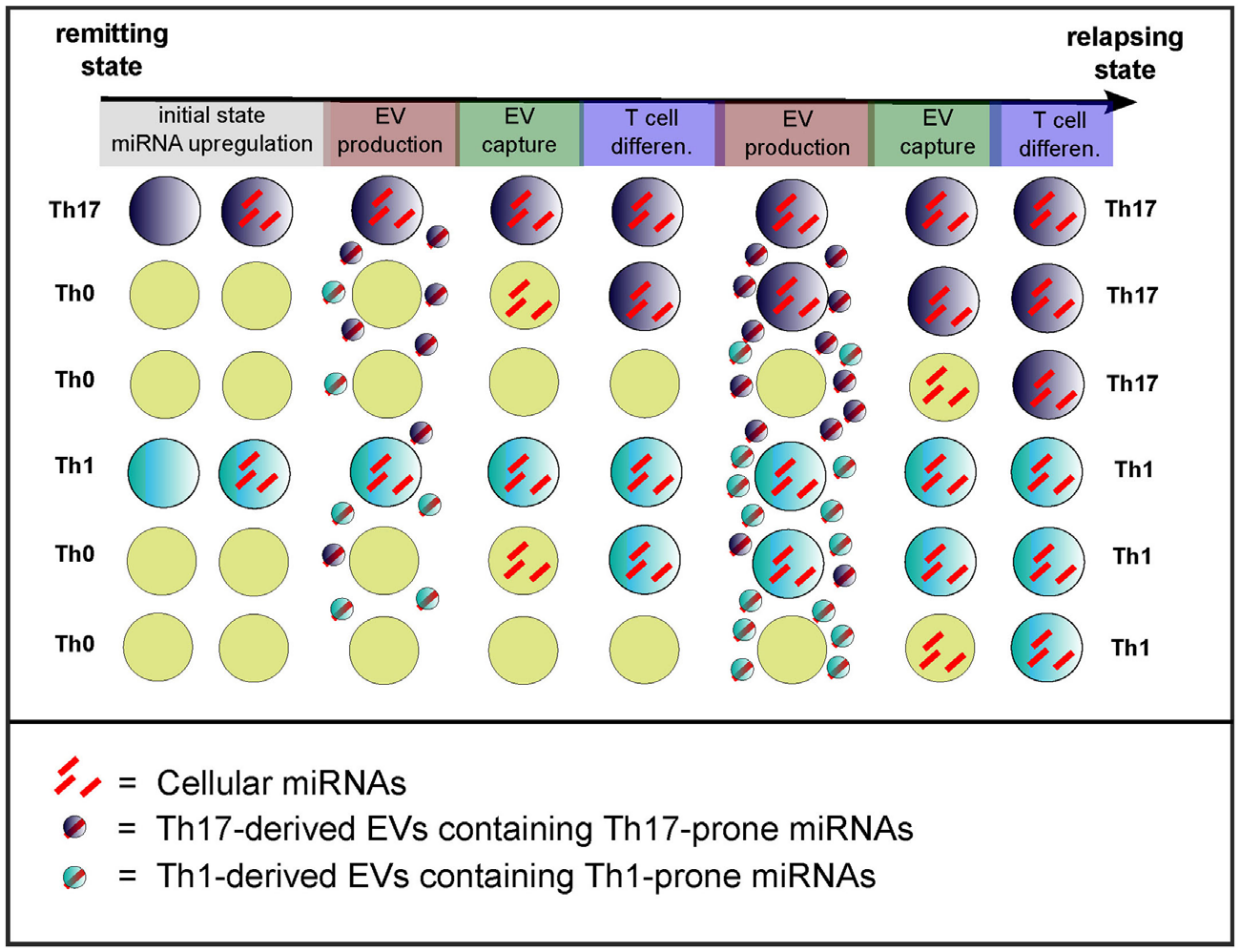
**Model of the cell non-autonomous effect of circulating miRNAs**. Exacerbated T cell differentiation (evidence being mainly for Th17 cells) might be a feature of the transition from remission to relapses (3). The initial state (remission) would depict poorly differentiated T cells, whereas it ends up during relapses with exaggerated T cell differentiation and activation. The transition from remission to relapses also correlates with increased dysregulation of miRNAs (3, 5) that would drive inflammatory T cell differentiation and activation. The model of cell non-autonomous effects of miRNAs consists in the transfer through EVs of T cell differentiation-driving miRNAs. The cause of initial miRNA dysregulation remains unknown. Light blue, dark blue, and yellow cells depict Th1, Th17, and non-differentiated Th0 cells, respectively.

The effect of miR-155 and miR-326 on MS pathogenesis is cell mediated, and their role when dysregulated in plasma has to be further investigated. Nevertheless, EV-associated miRNAs display the most plausible hypothesis to explain how a subset of cells with altered miRNA expression could generate an overall dysregulated network disrupting functional homeostasis during relapses (model of cell non-autonomous effect in Figure 4). Exacerbated T cell differentiation has not yet been shown to correlate in a restrictive manner with relapses. However, recent evidence suggest that both differentiation of Th17 cells (3) and infiltration into the CNS by Th17 cells (58) are increased in relapsing patients, as compared to remitting patients. Moreover, one can notice that (i) upregulation of cellular miR-326 in CD4^+^ T cells is observed in relapsing patients but not in remitting ones (3) and (ii) upregulation of both circulating miR-326 and miR-155 has been assessed in relapsing patients only (5). miRNA transfer from the cellular to the circulating compartment may thus constitute a putative trigger of the switch from remission to a relapsing state in MS patient. Based on the EV hypothesis, it is worth considering miRNA transfer between:

- T cells which could favor Th1 and Th17 differentiation and promote their activation (proposed in Figure 4) and
- periphery and CNS which could help to further comprehend the interactions between the CNS and the immune system.

The next section will focus on miRNAs as biomarkers for MS subtype and severity.

### Dysregulated Circulating miRNAs: New Biomarkers for MS?

The definitive diagnosis of MS is based on the McDonald Criteria to demonstrate the dissemination of CNS lesions in space and time in patients with symptoms suggestive of MS (59). It combines clinical examination (motor or sensory problems, optic neuritis, Lhermitt’s sign, etc.), magnetic resonance imaging (MRI) to detect multifocal CNS lesions and, if required, an electrophoresis from CSF (lumbar puncture) to reveal oligoclonal bands of IgG, which testify of infiltrating plasmocytes.

So, why are biomarkers for MS still arousing interest? Emerging biomarkers, such as circulating miRNAs, display several advantageous features in comparison with previous methods:

- the possibility to support the results of the clinical diagnosis distinguishing MS subtypes (PPMS, RRMS, etc.) and quantify MS severity,
- the fact that collecting blood miRNAs and measuring their expression is easy, poorly invasive, and cheap,
- the robustness of circulating miRNAs which are highly stable, and
- the accuracy using composite biomarkers rather than a single one.

The chronicity in MS pathology is the cause of repetitive relapses causing irreversible neurodegeneration. Depending on MS subtypes (PPMS or RRMS), medical care will be different. Thus, the possibility to anticipate MS evolution is crucial. Different statistical methods are used to predict the diagnostic accuracy of a biomarker: the area under the curve (AUC) (Box 1A) and the experimental sensibility/specificity (Boxes 1B,C). Interestingly, several studies analyzed specific circulating miRNAs differentially expressed according to MS subtypes (9, 18, 60).

Box 1 **Statistical tools to evaluate the relevance of a biomarker.**

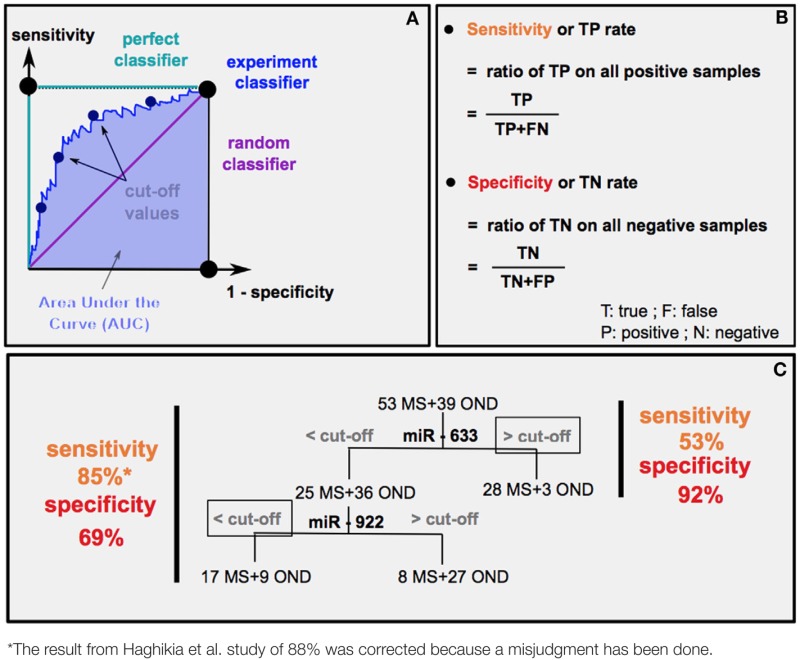

How can we estimate the diagnostic accuracy of miR-633 and miR-922 to distinguish MS from other neurological diseases (OND) (60)?

*First method*: receiver operating characteristic (ROC) curves **(A)** plot sensitivity (*y* axis) versus 1 − specificity in (*x* axis). An experiment good predictor curve will locate between a random classifier (50% chance predicting MS or OND) and a perfect classifier (100% chance predicting MS or OND). Calculation of the *area under the curve (AUC)* gives the level of *diagnostic accuracy*, which corresponds to the probability that random MS patients have higher (if upregulated) or lower (if downregulated) miRNA levels than OND ones.

*Second method*: it is possible to determine a *sensitivity* and *specificity* **(B)** score for each cut-offs (estimated threshold of miRNA level separating MS from OND), thus drawing the ROC curve. The highest sensitivity and specificity score indicates the most relevant *cut-off*. The example in **(C)** represents the result of sensitivity and sensibility (60) (*left*) and the predicted results using only miR-633 (*right*).

Fenoglio and colleagues (18) found out using the AUC method that blood circulating miR-223 and miR-15b could discriminate PPMS from healthy controls with a level of diagnostic accuracy of 80 and 75%, respectively. It was estimated that MRI clinical diagnosis in the onset of patients with clinically isolated syndrome (CIS) had 80% diagnostic accuracy predicting MS (61). It remains to be determined whether monitoring miRNA levels in CIS patients could help improving the accuracy of MRI-based diagnostic. Moreover, pathologies that mimic MS prevent clinical investigations from accurately predicting MS. Interestingly, a miRNA profiling study of the CSF of MS patients (60) (Box 1) reported the cut-off values of the expression of two miRNAs in order to discriminate MS from OND. They showed that the composite use of miRNAs is promising to gain diagnostic accuracy differentiating MS from OND (Box 1) but also distinguishing RRMS from PPMS entities. It remains to be determined whether circulating miRNAs could be used to assess or confirm the diagnosis of MS subtypes or even further subdivide MS subtypes into smaller clusters for the customization of treatments. It is also of interest to understand whether the variable efficiency of treatments according to MS subtypes (62) correlates with changes in miRNA levels, since several treatments were shown to impact miRNA levels (63–65). Besides, miRNA levels in plasma were shown to correlate with the expanded disability status scale of MS patients (9, 18). miRNA quantification could thus be used as a molecular marker to access MS severity. Moreover, miRNAs probably constitute reliable biomarkers, as they were demonstrated more stable than mRNAs and stabilized by their vectors (EVs or proteins) (8). Indeed, RNase treatments, freezing–thawing cycles, boiling temperature exposures, and pH ranges had no effect on miRNA stability in plasma (8).

Although its procedure is not the most convenient, the analysis of CSF miRNAs from MS patients is relevant considering that it may better reflect the level of brain damage. The authors mentioned the promising future of miRNAs as biomarkers of MS (60) but aptly considered its limitations at the moment. Profiling studies are indeed performed on relatively too small samples, not sufficient to attest that one miRNA could be a robust biomarker. Moreover, patients are chosen from a confined geographic area with higher similarities in their susceptibility genes. Further investigations about whether the expression of miRNAs could be used to biomark MS should be performed on larger samples. There is a need of standardization for miRNA extraction, quantification, and expression measure. Such a standardization would enable to compare studies and to define an optimal composite of miRNAs for biomarking MS.

## Discussion

There is current evidence that at least 60 circulating miRNAs would be dysregulated in MS patient’s blood and profiling results are continuously emerging. The current challenge relies on linking this network with the network of cellular dysregulated miRNAs. EVs are the most relevant miRNA carriers and could explain the relation between these two dysregulated networks. Both immune and neural cells, active actors of MS physiopathology, produce and receive EVs. This observation has to be considered besides the demonstrated implication of some cellular miRNAs, including miR-326 and miR-155, which were found to promote inflammation and demyelination.

Indeed, the hypothesis of miRNA-containing EVs raises the issue of a possible cell non-autonomous amplification of inflammation and demyelination through abnormal miRNA transfer. It has to be seriously considered during a period of relapse, in which symptom worsening is sudden. Interestingly, current treatments to prevent from worsening effects of relapses were shown to reduce miRNA dysregulation in peripheral blood mononuclear cells and T cells in particular (63–65), but an effect on circulating miRNAs has not been described yet. miRNA use as biomarkers is an attractive clinical tool. Circulating miRNAs are highly stable in blood, easy to collect, and the quantification method, if standardized, can be accurate and cheap. They are putative biomarkers to diagnose MS but could also serve differentiating MS subtypes, anticipating relapses and proposing a customized treatment. In clinical perspectives, much expectation is attributed to miRNAs for monitoring changes even before the pathology is developed. It applies for chronic diseases, such as MS, and various diseases, including inflammatory bowel diseases, xenobiotic-induced liver toxicity, and cancer.

## Author Contributions

FJ acquired the data and drafted the manuscript. ND revised the manuscript and made substantial contributions to its final content and design. All authors read and approved the final manuscript.

## Conflict of Interest Statement

The authors declare that the research was conducted in the absence of any commercial or financial relationships that could be construed as a potential conflict of interest.

## Abbreviations

BBB, brain blood barrier; CNS, central nervous system; CSF, cerebrospinal fluid; EAE, experimental autoimmune encephalomyelitis; EV, extracellular vesicle; MRI, magnetic resonance imaging; MV, microvesicle; MVBs, multivesicular bodies; OND, other neurological diseases; RISC, RNA-induced silencing complex.
